# Magnetic Resonance Imaging of Burdekin Plum During Fruit Development

**DOI:** 10.1002/fsn3.70707

**Published:** 2025-07-25

**Authors:** Gengning Chen, Yasmina Sultanbawa, Nyoman D. Kurniawan

**Affiliations:** ^1^ ARC Industrial Transformation Training Centre for Uniquely Australian Foods, Queensland Alliance for Agriculture and Food Innovation The University of Queensland Indooroopilly Queensland Australia; ^2^ Australian Institute for Bioengineering and Nanotechnology, Centre for Advanced Imaging The University of Queensland St Lucia Queensland Australia

**Keywords:** Burdekin plum, Diffusion Tensor Imaging, magnetic resonance imaging, relaxation, water content, Zero Echo Time

## Abstract

In recent years, magnetic resonance imaging (MRI) has been applied to non‐destructively study tissue changes during fruit development. In this study, MRI was utilized to investigate the structural characteristics of the fruit of *Pleiogynium timoriense* (Burdekin plum) at four development stages. Fruits were imaged using structural, relaxation, and diffusion MRI acquisition protocols at 9.4T. Results showed that MR images can differentiate major fruit tissues including mesocarp, endocarp, and seeds. Diffusion tensor imaging was able to visualize the water transportation pathways of the fruit. Quantitative imaging results showed that T1, T2, T2*, and ADC maps obtained by MRI reflected the structural differences between major fruit tissues (mesocarp, endocarp, seeds). Their values changed during fruit development, especially during the early stages. T1, T2, T2*, and ADC of endocarp and seed reduced significantly (*p* < 0.05) from stage 1 to stage 2, while that of mesocarp increased significantly (*p* < 0.05) from stage 1 to stage 2. Principle component analysis showed that T1 changes in mesocarp were correlated to the water content, and T2 changes were correlated to the ADC. This study confirms the utility of MRI for measuring tissue changes non‐invasively in Burdekin plum fruit development.

## Graphical abstract

MRI was used to distinguish major tissues in Burderkin plums, including mesocarp, endocarp, and seeds. Quantitative T1, T2, T2*, and ADC of these tissues changed during fruit development, with the most notable changes happening from stage 1 to stage 2. Diffusion MRI tractography was used to visualize the fruit water transportation pathways.

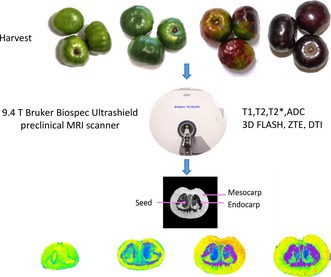

## 1 Introduction

*Pleiogynium timoriense* is a tropical rainforest tree native to Australia. Only one species of *Pleiogynium* is acknowledged in Australia (Jessup 1985). It is commonly known as Burdekin plum (BP) in Australia and is known for its fruits (Lake 2015). The fruits are traditionally consumed by Aboriginal people and subsequent settlers (Low 1991). Fossil evidence of *Pleiogynium* from the Oligocene has been found in Queensland, Australia, indicating the presence of *Pleiogynium* for over 30 million years, with its morphology remaining relatively unchanged (Rozefelds et al. 2014).

BP fruits have a unique shape and structure, resembling a flying saucer (Rozefelds and Kane 2016) with a slender flesh layer and a sizable stone. Like other fruits in the Anacardiaceae family, BP is drupaceous, and the pericarps can be distinguished into three layers, including a membranous exocarp, fleshy mesocarp, and woody endocarp (Herrera et al. 2018). Within the endocarp, it usually contains around 5 to 12 reniform locules in a radial arrangement. Each locule has a germination valve or opercula towards the distal end of the fruit for the seed to germinate (Wannan and Quinn 1990).

There is limited information about BP structural changes during fruit development. Over the course of fruit development, the tissues experience multiple morphological and compositional changes. Conventionally, these changes are usually studied using destructive methods such as histology and chemical analysis (Glidewell et al. 1999). BP fruit morphology and structure have been studied using traditional photography and histology (Wannan and Quinn 1990), and recently using X‐ray computed tomography (Rozefelds et al. 2014).

Magnetic resonance imaging (MRI) has been widely applied in clinical imaging, with increasing interest in its application to study plants in recent years (Borisjuk et al. 2012). MRI is an imaging technique based on nuclear magnetic resonance. MRI can provide a wide range of image contrasts for quantitative analysis, which complement ionizing imaging techniques such as X‐ray radiography and computed tomography. MRI generates images based on radiofrequency signals emitted from a sample when it is placed in an external magnetic field. The manipulation of the sample proton magnetic moments inside the scanner, by unique radiofrequency pulse sequences, is used to generate MR image contrasts (Weishaupt et al. 2006).

MR image contrast and signal intensity are influenced by several factors, including the strength of the magnetic field, the pulse sequence, and the sample proton density, proton relaxation, and diffusion (Weishaupt et al. 2006). After the application of a radiofrequency pulse, the excited spins return to their original state by the relaxation process. The relaxation process can be characterized by longitudinal relaxation (T1) and transverse relaxation (T2 and T2*). T1 describes the process of the spins returning to the original equilibrium state. T2 describes the decay of transverse magnetization due to spin–spin interaction. T2* describes the magnetization decay in the transverse plane from T2 relaxation and inhomogeneities of the external magnetic field (Callaghan et al. 1994). The apparent diffusion coefficient (ADC) obtained from the diffusion‐weighted sequence contains a combination of water diffusion rate, membrane permeability, cell size, and air cavity (Zhang and McCarthy 2012). Relaxation times and the diffusion coefficient reflect the biological properties of imaged tissue and are among the most important MRI parameters employed in quantitative MRI (Alzola‐Aldamizetxebarria et al. 2022).

In plant imaging, MRI typically depicts sample spin density distribution of mostly water, lipid, and sugar, and the relationship between the spins and their molecular environment (Ahmed et al. 2017). Fleshy fruits are abundant in water and sugar. MRI has been valuable in analyzing various aspects of fruits, including determining water content, distribution, and mobility (Patel et al. 2015), elucidating internal structure (Musse et al. 2009), water transportation (Windt et al. 2006) and metabolites (Musse and Van As 2018), differentiating geographical origins (Sequi et al. 2007) and seasonality (Ciampa et al. 2010), monitoring changes during fruits maturation and ripening (Callaghan et al. 1994) and identifying qualities such as voids, bruises, watercore, and drying (Chen et al. 1989; Defraeye et al. 2013).

MRI signal intensity, relaxation times, and diffusion coefficient have been widely used to study plant anatomy and physiology (Musse and Van As 2018; Van As 2007). Quantitative MRI has been applied in various fruits to study fruit development such as mango, kiwifruit, persimmon, pomegranate, and grapes (Srivastava et al. 2018). One recent example is their use to study an Australian native fruit, the green plum, during maturation (Fyfe et al. 2023). MRI signal intensity, oil and water resonance peaks ratio, and proton T1 and T2 relaxation have been found to correlate with avocado maturity (Chen et al. 1993). Similarly, T2 relaxation and ADC have also been found to be suitable indicators of tomato maturity (Zhang and McCarthy 2012). The mean signal intensity has been used to differentiate three major parts of cherry tomato (pericarp, locule and placenta) and monitor their changes from green to red stages (Seunghoon et al. 2020). In persimmon, T1 relaxation of its mesocarp and vascular tissue showed a sigmoidal growth during fruit development, while T2 remained steady (Clark and MacFall 2003).

In this paper, we investigated physical and chemical changes during the development of BPs using MRI, by measuring T1 and T2 relaxation and diffusion coefficient evolution and tissue water content during the fruit development. We also demonstrated the use of 3D Fast Low Angle Shot, Zero Echo Time (ZET), and Diffusion Tensor Imaging (DTI) to examine distinct structural changes which accompanied fruit development.

## 2 Material and Methods

### 2.1 Samples

BPs were collected in Brisbane (Queensland, Australia) in 2022. There were four fruit development stages studied, consisting of the immature green stage (stage 1), the mature green stage (stage 2), the turning stage (stage 3) and the dark maroon stage (stage 4). The samples included 2 fruits each from 3 trees for each of the fruit development stages (i.e., at total of 6 fruits per maturation stage).

### 2.2 Magnetic Resonance Imaging

MRI were acquired using a 9.4T (30 cm) Bruker Biospec Ultrashield preclinical MRI scanner (Bruker, Ettlingen, Germany), equipped with a BGA 12S HP imaging gradient (Gmax 660 mT/m) and using a 40 mm quadrature coil. Freshly harvested fruit was wrapped in dry tissue paper, placed in the coil, and imaged at room temperature 22°C. The MRI acquisition parameters are shown in Table 1, and the complete Bruker MRI protocols are available at https://doi.org/10.48610/57ebc66. Data acquisition and processing used ParaVision 6.0.1 (Bruker, Ettlingen, Germany).

**TABLE 1:** MRI sequence parameters for imaging the Burdekin plums.

Item	Sequence	Echo time (TE) (ms)	Repetition time (TR) (ms)	Flip angle (degree)	Field of view (mm)	Matrix size (read × phase × slice)	Resolution (read × phase × slice mm)	Number of excitations	Scan time
T1	2D Rapid Acquisition with Relaxation Enhancement and Variable Repetition Times (RAREVTR)	8.5 RARE factor = 2	427–5500 (6 TRs)	90, 180	50 × 50	512 × 256 × 24	0.1 × 0.2 × 1.0	1	24 min 51 s
T2	2D Multi‐Slice Multi‐Echo (MSME)	8–240 (30 echoes)	4100	90, 180	50 × 50	256 × 256 × 24	0.2 × 0.2 × 1.0	1	17 min 29 s
T2*	2D Multi‐Echo Gradient echo (MGE)	2.4–37.4 (8 echoes)	800	50	50 × 50	256 × 256 × 24	0.2 × 0.2 × 1.0	2	5 min 7 s
ADC	2D Spin echo Diffusion Weighted Imaging (DWI): 1 *b* = 0, 6 directions with *b* = 500 s/mm^2^	16.5	1000	90	50 × 50	256 × 128 × 24	0.2 × 0.4 × 1.0	1	11 min 12 s
Morphology	T1‐weighted 3D Fast Low Angle Shot (FLASH)	3.2	30	30	45 × 45 × 45	450 × 225 × 225	0.1 × 0.2 × 0.2	1	25 min 18 s
Morphology	Zero Echo Time (ZTE)	0	3.840	2.5	50 × 50 × 50	256 × 256 × 256	0.2 × 0.2 × 0.2	1	13 min 13 s
DTI	3D DWI: 2× *b* = 0 images, and 15 diffusion encoding directions with *b* = 500 s/mm^2^ (δ/Δ = 2.5/8.5 ms)	15.6	250	90, 180	45 × 45 × 45	128 × 128 × 128	0.35 × 0.35 × 0.35	1	14 h 30 min

T1, T2, T2* and ADC were acquired using two dimensional (2D) Rapid Acquisition with Relaxation Enhancement with Variable Repetition Times (RAREVTR), 2D Multi‐Slice Multi‐Echo (MSME), 2D Multi‐Echo Gradient Echo (MGE), and 2D Spin Echo Diffusion Weighted Imaging (DWI), respectively. Three separate regions of interest (ROI) from each fruit, including the mesocarp, endocarp, and seed on the corresponding center slice image (Figure S1) were manually segmented for quantitative MRI analysis. Relaxation times (T1, T2, T2*) and ADC were obtained from these ROIs by fitting the image intensity into T1 saturation recovery, T2 exponential decay, and diffusion exponential decay equations (Westbrook 2016).

High‐resolution three‐dimensional (3D) images were acquired using 3D Fast Low Angle SHot (FLASH) (Frahm et al. 1986) and ZTE (Weiger et al. 2012). The 3D images were acquired with isotropic image resolutions to reduce partial‐volume effect from 2D imaging with thick slices and allow examination of fine structures. The T1‐weighted 3D FLASH sequence was selected to produce high‐resolution images with a relatively short acquisition time. A TE of 3.2 ms, which was the shortest time achievable, was selected to minimize susceptibility artifact from the endocarp and close to the in‐phase water/fat cycling period at 9.4T (~0.75 ms). While the FLASH sequence was useful to visualize young whole fruits (stage 1), it could not be used for the later stages as the endocarp lignification resulted in dramatically shortened T2*. Therefore, the ZTE sequence was performed to solve this problem and regain the signals. 3D animation of maximum intensity projection (MIP) of 3D FLASH and ZTE images (Video S3) was created using the program Horos (horosproject.org).

DTI data were acquired using 3D DWI Spin Echo with 15 diffusion encoding gradients and 2 *b* = 0 images. The number of diffusion directions and image resolution were selected to fit the time available for an overnight acquisition. A *b*‐value of 500 s/mm^2^ was selected from a range of *b*‐values (250–800 s/mm^2^) that were successfully used in the DTI study of seedless grapes at 11.7 T (Dean et al. 2014; Gruwel et al. 2013). DTI was used to visualize the fruit fibrous structures, as they were invisible from the other MR modalities. Fiber tractography was reconstructed using deterministic Fiber Assignment by Continuous Tracking (FACT) algorithm using the program DiffusionToolkit/TrackVis (TrackVis.org). The fiber tracks (Video S4) were exported to H.264 format using the Apple QuickTime player program (version 10.5).

### 2.3 Water Content

Water content of fruit flesh (including mesocarp and exocarp) was analyzed by Association of Official Analytical Collaboration (AOAC) method 925.10 (AOAC 2000). Briefly, for each fruit, around 1 g BP flesh was separated from the stone using a knife, then the flesh was weighed into an aluminum dish and dried at 105°C for 16 h using a convection oven (Steridium dg‐160, Brisbane, Australia).

### 2.4 Data Analysis

Data analysis was conducted using XLSTAT (Addinsoft 2022, Paris, France). ANOVA was applied to test the differences between samples with Tukey–Kramer honestly significant difference (HSD) for pairwise comparison (*p* < 0.05). A principal component analysis (PCA) was conducted on the mean water content and quantitative MRI results to explore sample associations. Data were standardized based on Pearson correlation in PCA analysis.

## 3 Result and Discussion

### 3.1 Visualization of BP Structures Using MRI

**FIGURE 1:**
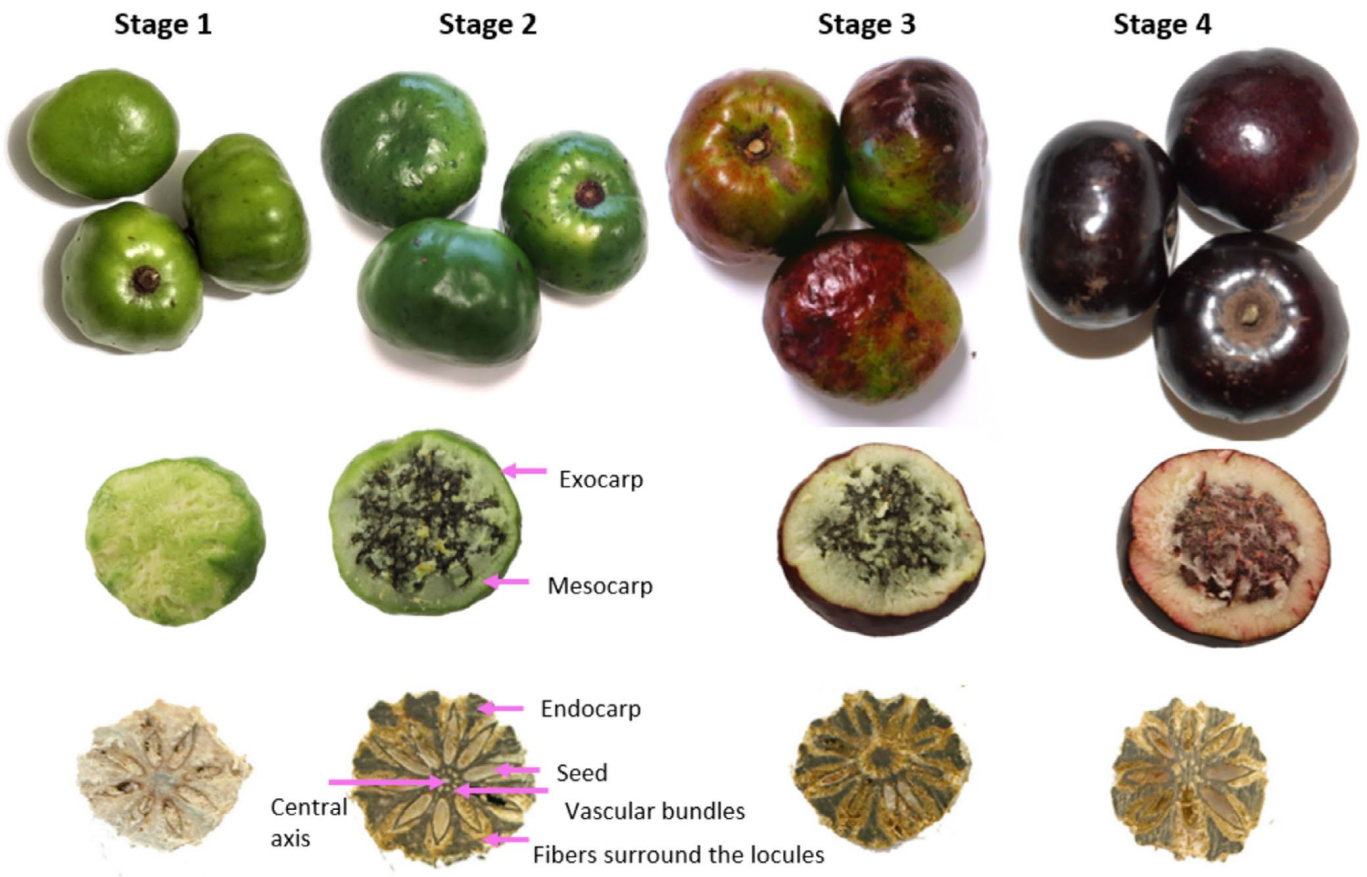
Photography of Burdekin plum at four fruit development stages. Top: Whole fruit; Middle: Fruit with partial removal of mesocarp; Bottom: Sliced fruit stone.

Our interpretation of mesocarp and endocarp (Figure 1) follows that of Herrera et al. (2018), as these two regions were discernible in the MR images. The other description of BP structures (Rozefelds et al. 2014; Wannan and Quinn 1990) assigned the fibrous tissue under the flesh as inner mesocarp instead of endocarp.

**FIGURE 2:**
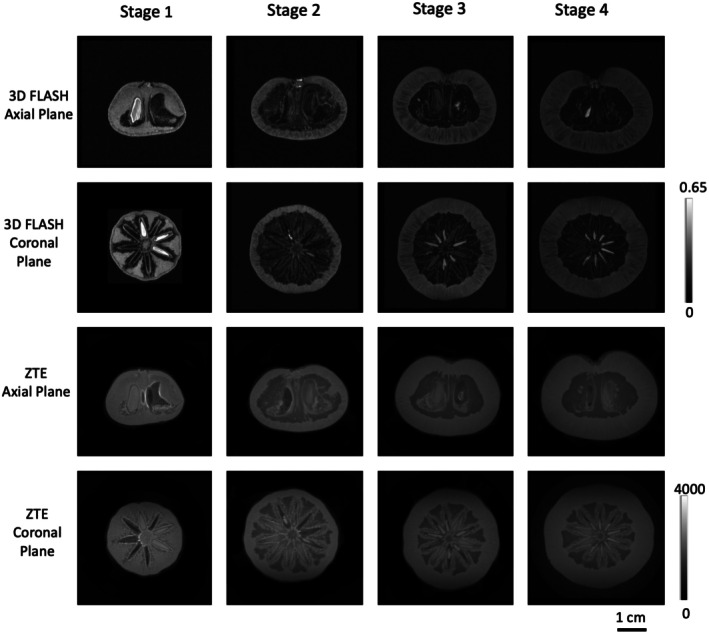
Axial and coronal plane of 3D FLASH and ZTE. MR image slices were shown for the middle slice of Burdekin plum. The gray scale maps indicate image intensities in arbitrary unit.

Gradient echo (FLASH) and ZTE MR images of the BPs are shown in Figure 2 and Video S3. In coronal plane MR images, the central axis with surrounding vascular bundles was seen, especially after stage 1 as the endocarp lignified, leading to the widening distance between the vascular cavities and central axis. In axial plane MR images, the basal end of BP became more depressed as the exocarp expanded during maturation, while the apical end started as being slightly concave and gradually became rounded. The increase in fruit dimensions was also clearly observed during their development. Structural features revealed non‐destructively by MRI agreed with the photograph of BP in Figure 1 and from literature (Herrera et al. 2018).

The exocarp, which comprises a thin layer of parenchyma (Wannan and Quinn 1990), was not discernible in the current MRI experiment due to limitations in image resolution (van Schadewijk et al. 2020). BP mesocarp comprises a layer of parenchymatous cells (Herrera et al. 2018), which elongate as the fruit develops. After stage 1, the mesocarp was clearly seen in MRI as its thickness increased and the endocarp became darker. The interspersed air pockets, which appeared as alternate dark strips in the mesocarp, were also seen more clearly.

At stage 1, the endocarp, which is composed of parenchyma and sclerenchyma, was less distinguishable in the MRI from the thin mesocarp. As the sclerenchyma becomes lignified when the BP is mature (Wannan and Quinn 1990), the endocarp appeared dark and easily distinguishable from the mesocarp in both 3D FLASH and ZTE images. The endocarp could be differentiated from the mesocarp using curved striations of the endocarp seen in the axial plane images and using the hypointense (dark) lines seen in the coronal plane of the 3D FLASH image.

The reniform locules were evident especially in the axial plane of ZTE images, and the radial arrangement of multiple locules, with some containing seeds, was evident particularly in the coronal plane of ZTE images. Post stage 1, the seeds became less visible in the 3D FLASH images compared to that in the ZTE images. This was caused by reduced MRI signal from drying seeds and increased air content in the endocarp. Typically, the tissue–air pocket interfaces produce magnetic field gradients causing increased field inhomogeneities and rapid loss of spin magnetization in the transverse plane (i.e., short T2 and T2* relaxations). Such signal loss is especially significant in gradient echo (FLASH) images (Musse and Van As 2018). The ZTE sequence is useful to overcome the difficulty in imaging samples with very short transverse relaxation times, such that for imaging solid samples with low water content (Garwood 2013; Tuomainen et al. 2022). As the mesocarp became more hydrated as the fruit matured (increased T1, T2 and ADC shown in Figure 4), small water exchanges between the endocarp/mesocarp structures could contribute to the proton signals in the endocarp shown by ZTE.

Using ZTE, the visibility of the overall fruit structures was enhanced. The locules could be differentiated based on whether they were empty (hypointense), contained seeds (isointense), or were filled with fluid (hyperintense). In comparison, empty locules and locules with seeds appeared hypointense in the 3D FLASH images. The seed coats became obvious from stage 2 onwards, as they dried out during maturation. The tortuous fiber tracts surrounding the locules were also prominent. Using MR spectroscopy, bright areas inside the locules were confirmed to be filled with fluid and not seeds, as they appeared bright in coronal spin‐echo T2‐weighted images (Figure S2). Such hyperintense fluid‐filled locules have also been observed in persimmons (Clark and MacFall 2003) and Scots pine seeds (Tuomainen et al. 2022).

### 3.2 MR Relaxation in BP

**FIGURE 3:**
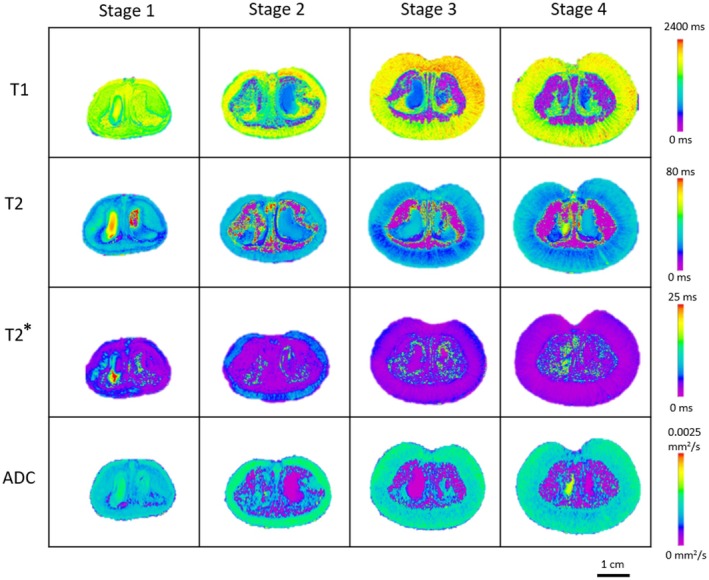
Calculated T1, T2, T2* and ADC maps. MR image slices were shown for the middle slice of Burdekin plum. Colour scale maps indicate image intensity in relation to relaxation times (ms) and diffusion coefficient (mm^2^/s).

T1, T2, and T2* maps of the BP are shown in Figure 3. Three ROIs were drawn on the mesocarp, the endocarp, and the seed. The mesocarp was distinguishable from the endocarp and the seeds as the mesocarp had relatively higher T1 and lower T2 values, which indicated a higher water content (MacFall and Johnson 1994).

**FIGURE 4:**
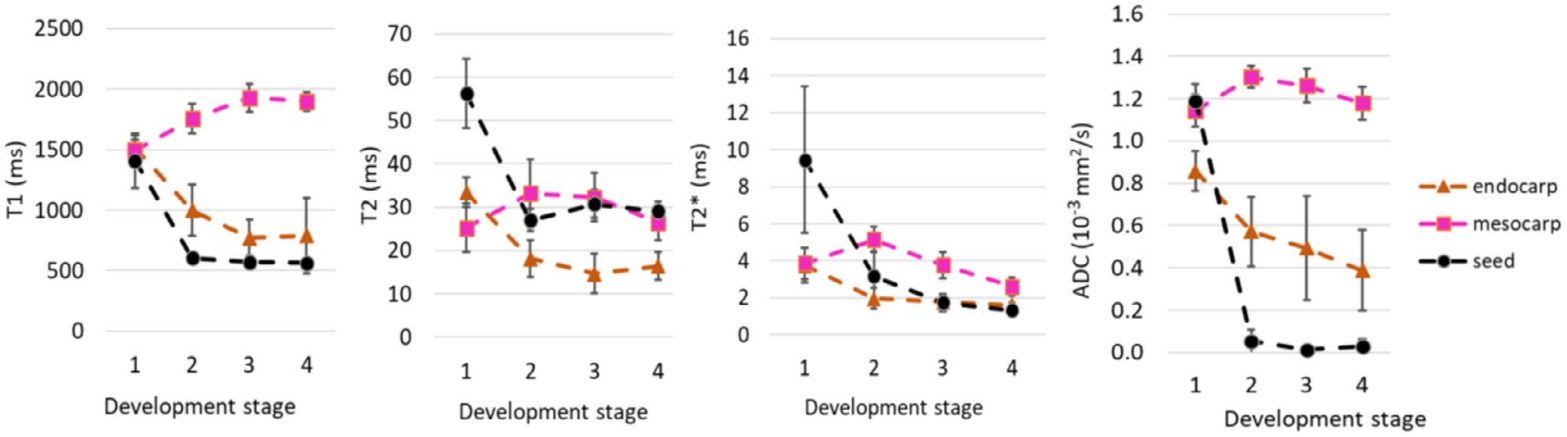
T1, T2, T2* and ADC of Burdekin plum measured at four development stages. Graphs represent the mean and standard deviation at each development stage.

Most significant changes in the BP MR relaxation parameters occurred during development from stage 1 to stage 2 (Figure 4). The relaxation times increased in mesocarp (T1: 1494 ± 125 to 1754 ± 122 ms (*p* < 0.05), T2: 25 ± 6 to 33 ± 8 ms) and decreased in endocarp (T1: 1517 ± 65 to 997 ± 211 ms (*p* < 0.05), T2: 33 ± 3 to 18 ± 4 ms (*p* < 0.05)) and seeds (T1: 1408 ± 224 to 603 ± 19 ms (*p* < 0.05), T2: 56 ± 8 to 27 ± 3 ms (*p* < 0.05)).

The increase in T2 of mesocarp has been reported in peach development (Musse et al. 2021). The increases in T1 and T2 have also been observed in Japanese pears during development (Geya et al. 2013). An MRI study of persimmon at 2 T showed that the mesocarp T1 grew sigmoidally from ~1000 ms (fruitlets) to ~2000 ms at commercial harvest, whereas its T2 increased slightly from ~80 ms (fruitlets) to ~100 ms at the second stage, then remains stable throughout maturation (Clark and MacFall 2003).

Numerous factors can affect proton relaxation; these encompass changes in cell size and structure, chemical composition, and magnetic susceptibility (Clark et al. 1997). The increase in mesocarp relaxation times has been attributed to the growth in cell size during fruit development (Musse et al. 2021). T2 is more sensitive than T1 in differentiating water compartments and mobility (Van As 2007), and T2 is also shorter in fructose and glucose than in sucrose (Clark et al. 1998). We observed that T2 of BP mesocarp had a decreasing trend from stage 2 (33 ± 8) to stage 4 (26 ± 4 ms), which could be attributed to increased proton exchanges among water and other molecules, including sugars and lipids (Van As 2007).

Transverse relaxation is also affected by molecular diffusion and internally generated magnetic field gradients due to the presence of air space between cells (Hills and Duce 1990). In BP mesocarp, this effect was exemplified by the short T2* and the reduction in T2* from stage 2 (5.1 ± 0.7) ms to stage 4 (2.6 ± 0.5 ms, *p* < 0.05). The presence of larger air space at later maturity stages made the T2* even shorter, since T2* is most susceptible to magnetic inhomogeneities (Musse et al. 2021). T2* values of BP mesocarp (1.9–6.4 ms) were similar to those reported for apples (~3 ms) (Werz et al. 2011).

The relaxation times in BP endocarp shortened as the fruit was maturing. This may be explained by reduced water mobility and water content due to endocarp lignification (Musse et al. 2021). In the seeds, water mobility and water content generally reduce as the seeds mature. For example, cherry seeds' T1 reduced from around 2 s when immature to 0.5 s when they matured (Ishida et al. 1997). Accumulation of paramagnetic ions (Goodman et al. 1996) as well as the formation and solidification of lipids in seeds (Glidewell et al. 1999) may also contribute to the shorter relaxation times in seeds.

### 3.3 Diffusion Changes in BP

Most notable changes in ADC also occurred from stage 1 to stage 2 (Figure 4). From stages 1 to 2, the ADC increased in the mesocarp (1.1 ± 0.075 to 1.3 ± 0.052 × 10^−3^ mm^2^/s, *p* < 0.05) and decreased in the endocarp (0.86 ± 0.095 to 0.57 ± 0.16 × 10^−3^ mm^2^/s, *p* < 0.05) and in the seeds (1.2 ± 0.080 to 0.05 ± 0.056 × 10^−3^ mm^2^/s, *p* < 0.05). Subsequently, the ADC remained relatively stable between stages 2 and 4. The initial increase in mesocarp ADC may be explained by cell size enlargement, making water molecules take a longer time to reach a barrier (Duval et al. 2005). The increase in ADC has also been observed in strawberry development (Goodman et al. 1996). Lower ADC of the BP mesocarp at stage 4 compared to the earlier stages could be attributed to a higher concentration of photosynthates, such as glucose and fructose, which accumulated during fruit development. This accumulation increased the tissue viscosity, and thus reduced the mobility of water (Ishida et al. 1997; Raffo et al. 2005). Also at this stage, the effect of the increase in photosynthates was suggested to outweigh the effect of the cell size and structure changes to the ADC values (Clark et al. 1998).

The ADC of BP mesocarp (1–1.3 × 10^−3^ mm^2^/s) is comparable to that of banana, cherry, and grape flesh (~1 × 10^−3^ mm^2^/s), which is lower than pure water (~2.3 × 10^−3^ mm^2^/s at 25°C) (Ishida et al. 1997; Raffo et al. 2005). The ADC values in fruit are reduced compared to free water diffusion, as water diffusion in the fruit tissue is hindered by cell membranes and other cellular content (Dean et al. 2014).

### 3.4 Water Content

The water content of developing BP flesh was lowest at stage 1 (79.6% ± 1.3%), then increased at stage 2 (81.9% ± 1.6%, *p* < 0.05) and remained stable at the later stages (stage 3 = 80.9% ± 1.6% and stage 4 = 82.1% ± 1.6%). The increase in water content has also been observed in other fruits during development (Chen et al. 2020; Frenkel and Hartman 2012).

**FIGURE 5:**
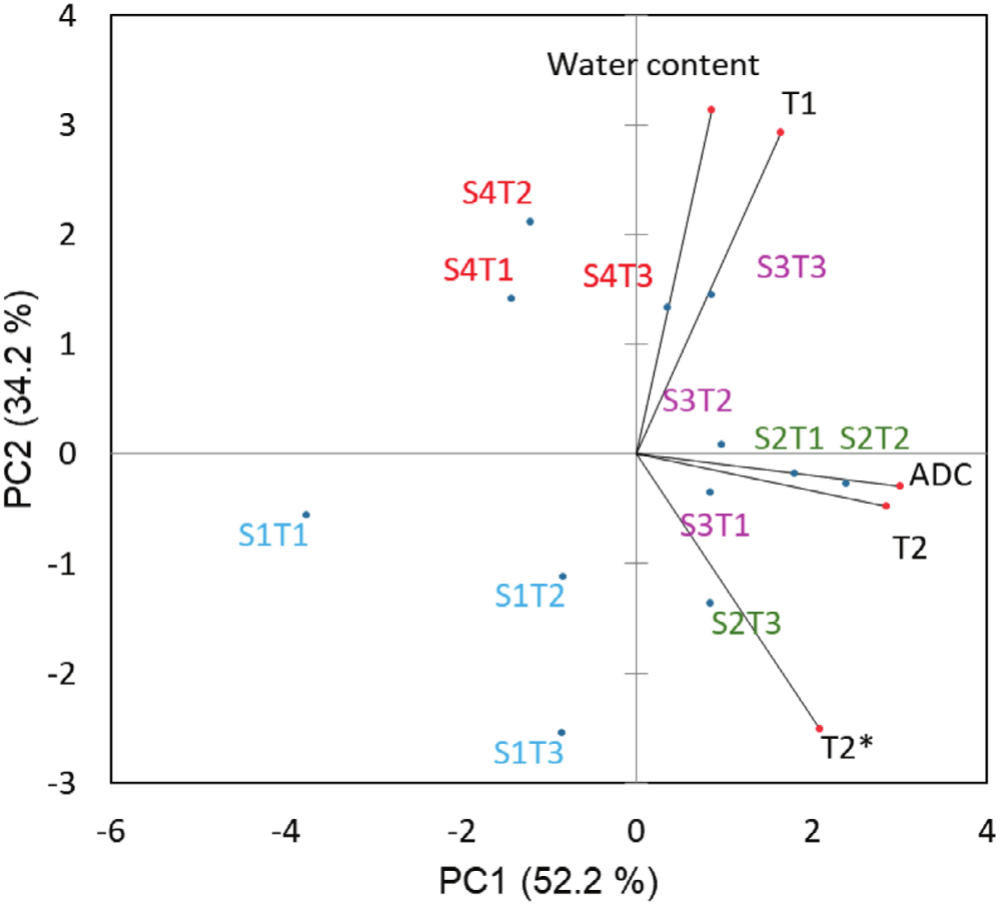
PCA biplot of BP water content and MRI parameters. Measurements were taken from BP mesocarp during fruit development. S1–S4 indicates the four stages of fruit development, T1–T3 indicates the three source trees.

A PCA was applied to the mesocarp water content and quantitative MRI parameters to explore their relationships. PCA showed that the first two principal components (PC1 and PC2) accounted for 86.4% of overall variance (Figure 5). ADC and T2 were heavily loaded on the positive side of PC1 (factor loading was 0.97 and 0.92, respectively), with a strong correlation (*r* ^2^ = 0.852, *p* < 0.05). Water content and T1 were heavily loaded on the positive side of PC2 (factor loading was 0.82 and 0.77, respectively), with a correlation (*r* ^2^ = 0.608, *p* < 0.05).

Although variation existed within each development stage, samples from the same development stage tended to cluster together. Stage 1 samples (located at the negative side of PC1 and PC2) were separated from samples from the later developmental stages. Stage 1 samples had the lowest water content and the lowest score on PC2. Stages 2 and 3 samples had relatively higher T2 and ADC (located at the positive side of PC1), while stages 1 and 4 samples had lower T2 and ADC values (located at the negative side of PC1). Stage 4 samples had the highest water content and had the highest scores on PC2, followed by stages 3 and 2 samples. These results indicated that a combination of these variables could discriminate samples at different development stages.

The contrast in MR images of plants primarily relies on diffusion and relaxation properties of water, which are largely influenced by proton exchange among different compartments and interaction with components including lipids, carbohydrates, and proteins (Van As and van Duynhoven 2013). The relaxation times can also be attributed to specific compartments in plant tissue. Water in vacuoles exhibits the longest relaxation times, followed by cytoplasm or extracellular space. In these compartments, diffusive exchange leads to single exponential behavior. On the other hand, proton exchange across the membrane influences the observed relaxation times, resulting in distinct T1 and T2 values. Differences in T2 among compartments are usually more pronounced than T1; therefore, T1 results have a more direct correlation with water content, and T2 results can better discriminate different cell compartments (Van As 2007). Such characteristics may explain our observation where the water content is better correlated with T1 rather than T2. Both T2 and diffusion coefficient can serve as indicators of molecular mobility (Qiao et al. 2005), highlighting our observation for their correlation.

### 3.5 DTI Tractography of Maturing BP Fruit

**FIGURE 6:**
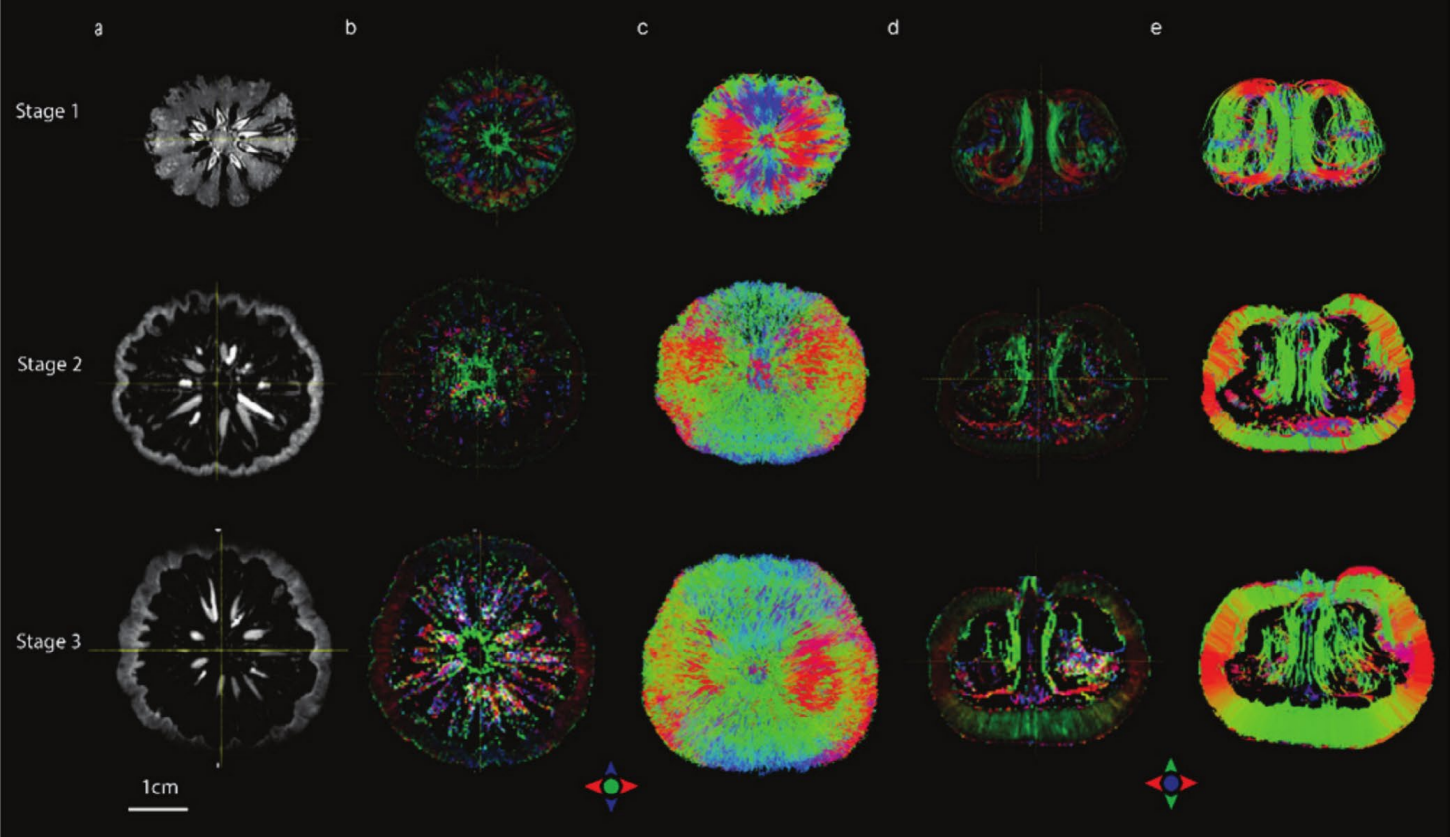
DTI tractography of BP from stage 1 to stage 3. (a) *b* = 0 images, taken at the middle coronal plane of the fruit. (b, d) Directionally encoded color fractional anisotropy maps taken at the middle of the fruit coronal and axial planes, respectively. (c) DTI tractography showing the water diffusion pattern seen from the stem end of the fruit. (e) DTI fibers shown as cross‐sections taken at the middle plane of the fruit. Red, green and blue color indicate principal direction of water diffusion in left–right, up‐down and in‐out of the viewing plane. Scale bar = 1 cm.

DTI can be used to reveal fruit water transport pathways (Dean et al. 2014). DTI measures the principal direction of water diffusion (tensor) in each voxel, where the degree of restricted diffusion is quantified as fractional anisotropy (FA) (Gruwel et al. 2013). In BP fruit, DTI revealed unique structures that were not visible in the conventional T1/T2‐weighted imaging (Figure 6), where directionally encoded color FA maps (red, green, blue) show the principal directions of water diffusion in each voxel (Figure 6b,d). The seeds were not clearly visible in DTI in both young and semi‐mature fruits.

During fruit development, the growing tissues of the fruit receive water and nutrients through a vascular network (Gruwel et al. 2013). At stage 1, the color FA maps showed the BP endocarp contained tissues that were packed in distinct orientations, with long vasculature projected from the central axis (green fiber tracks). These vascular tissues distributed radially as peripheral vasculatures (red/blue fiber tracks at the top and bottom of the fruit) and vertically around the fruit (green fiber bundles in the endocarp) and reconnected to the central axis (Figure 6c,e, Video S4).

As BP mature, the water transport in the mesocarp that radiated outward became more apparent as the cells became elongated. The tissues in the endocarp became less visible as it lignified, making the boundary between endocarp and mesocarp more discernible. The exocarp water paths could be observed being aligned orthogonally to the mesocarp water paths.

### 3.6 Study Limitation

The relaxation times and diffusion coefficient measured were averaged from all protons within each ROI. Therefore, these values are between the corresponding values for water and other proton‐containing molecules, such as lipids (Glidewell et al. 1999). However, multimodal MRI requires protracted scan times, which pose clear barriers to routine or field‐based studies. It has been found that despite MRI showing clear differences among fruit parts during fruit development, no consistent association between metabolites and relaxation parameters has been found (Capitani et al. 2010; Clark et al. 1998). Our interpretation of the observed changes in the MR parametric changes during the maturation of the BP should be taken cautiously because of the intricate nature of the factors affecting MRI during fruit development (Musse et al. 2021). The interpretation of results could be strengthened by histological and compositional analyses. Integrating such complementary methods in future work would enhance the reliability and interpretability of the MRI‐based observations. DTI were not performed in more fruit samples due to the very long scan time. Lastly, the generalizability of the findings is also constrained by the small number of samples. Future studies could include more samples to capture a wider range of variability.

### 3.7 Future Study

In this research, we focused on the four developmental stages of BPs growing in trees. The fruit is typically harvested at stage 4, with ripening and senescence occurring during storage. Future investigations to characterize the post‐harvest senescent stage will provide a more comprehensive understanding of fruit structural changes throughout the entire process, including post‐ripening structural degradation of the flesh.

## 4 Conclusion

Three major parts of the BP, including mesocarp, endocarp, and seeds, were visible via MR images, particularly through the ZTE images. Most notable changes of these three ROIs in relaxation times (T1, T2 and T2*) and ADC appeared when BP turned from immature (stage 1) to mature green (stage 2). The PCA result revealed the water content was correlated with T1. Further, the water distribution characteristics and their changes in BP tissue structure during development were visible via the 3D DTI tractography.

## Author Contributions

**Gengning Chen:** conceptualization (equal), data curation (equal), formal analysis (lead), investigation (lead), methodology (equal), project administration (equal), resources (equal), visualization (equal), writing – original draft (lead), writing – review and editing (lead). **Yasmina Sultanbawa:** conceptualization (equal), funding acquisition (lead), project administration (supporting), resources (equal), supervision (lead), writing – review and editing (supporting). **Nyoman D. Kurniawan:** conceptualization (equal), investigation (supporting), methodology (lead), project administration (supporting), resources (lead), software (lead), supervision (supporting), visualization (lead), writing – review and editing (lead).

## Conflicts of Interest

The authors declare no conflicts of interest.

## Supporting information


**Figure S1:** fsn370707‐sup‐0001‐FigureS1.docx.

**Figure S2:** fsn370707‐sup‐0002‐FigureS2.docx.

**Video S1:** fsn370707‐sup‐0003‐VideoS1.pptx.

**Video S2:** fsn370707‐sup‐0004‐VideoS2.pptx.

## Data Availability

The data that support the findings of this study are available within the article and/or its Supporting Information.
