# Supplementary material for: Magnetic Resonance Imaging of Burdekin Plum During Fruit Development
Source: Food Sci Nutr. 2025 Jul 25;13(7):e70707. doi: 10.1002/fsn3.70707 (PMC12290480; doi:10.1002/fsn3.70707)
Supplement: Supplementary file 1 — Figure S1: fsn370707‐sup‐0001‐FigureS1.docx. [file FSN3-13-e70707-s002.docx]

**Supplementary Materials**


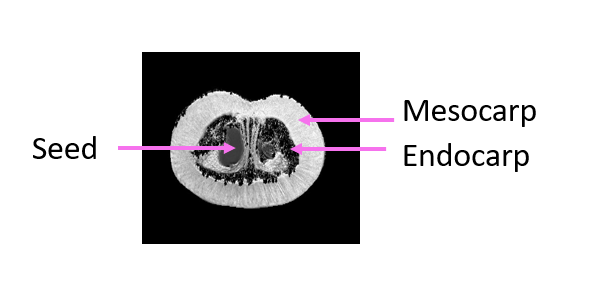


**Figure S1. MRI segmentation of Burdekin fruit tissue.** Three separate regions of interest (ROI) from mesocarp, endocarp and seed were manually drawn.
