# Supplementary material for: Magnetic Resonance Imaging of Burdekin Plum During Fruit Development
Source: Food Sci Nutr. 2025 Jul 25;13(7):e70707. doi: 10.1002/fsn3.70707 (PMC12290480; doi:10.1002/fsn3.70707)
Supplement: Supplementary file 2 — Figure S2: fsn370707‐sup‐0002‐FigureS2.docx. [file FSN3-13-e70707-s004.docx]

Supplementary Materials


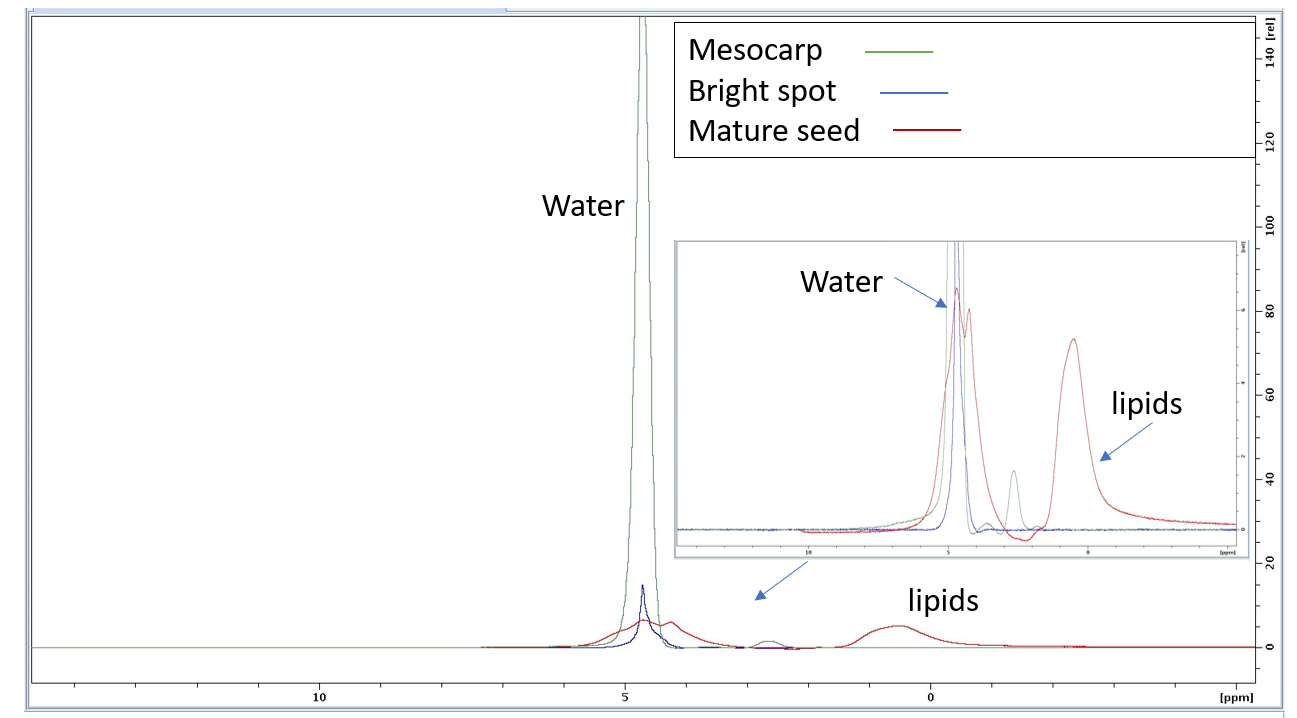


**Figure S2. MR spectroscopy from a mature fruit**. Data were acquired using a PRESS sequence, with the parameters TR/TE = 2000/20 ms, NEX=64, spectral width = 50 kHz, acquisition time of 11 mins per scan. Spectra were acquired using variable voxels placed in the seed, bright area in the locule and in the mesocarp.
