# Supplementary material for: Magnetic Resonance Imaging of Burdekin Plum During Fruit Development
Source: Food Sci Nutr. 2025 Jul 25;13(7):e70707. doi: 10.1002/fsn3.70707 (PMC12290480; doi:10.1002/fsn3.70707)
Supplement: Supplementary file 4 — Video S2: fsn370707‐sup‐0004‐VideoS2.pptx. [file FSN3-13-e70707-s001.pptx]

## Slide 1
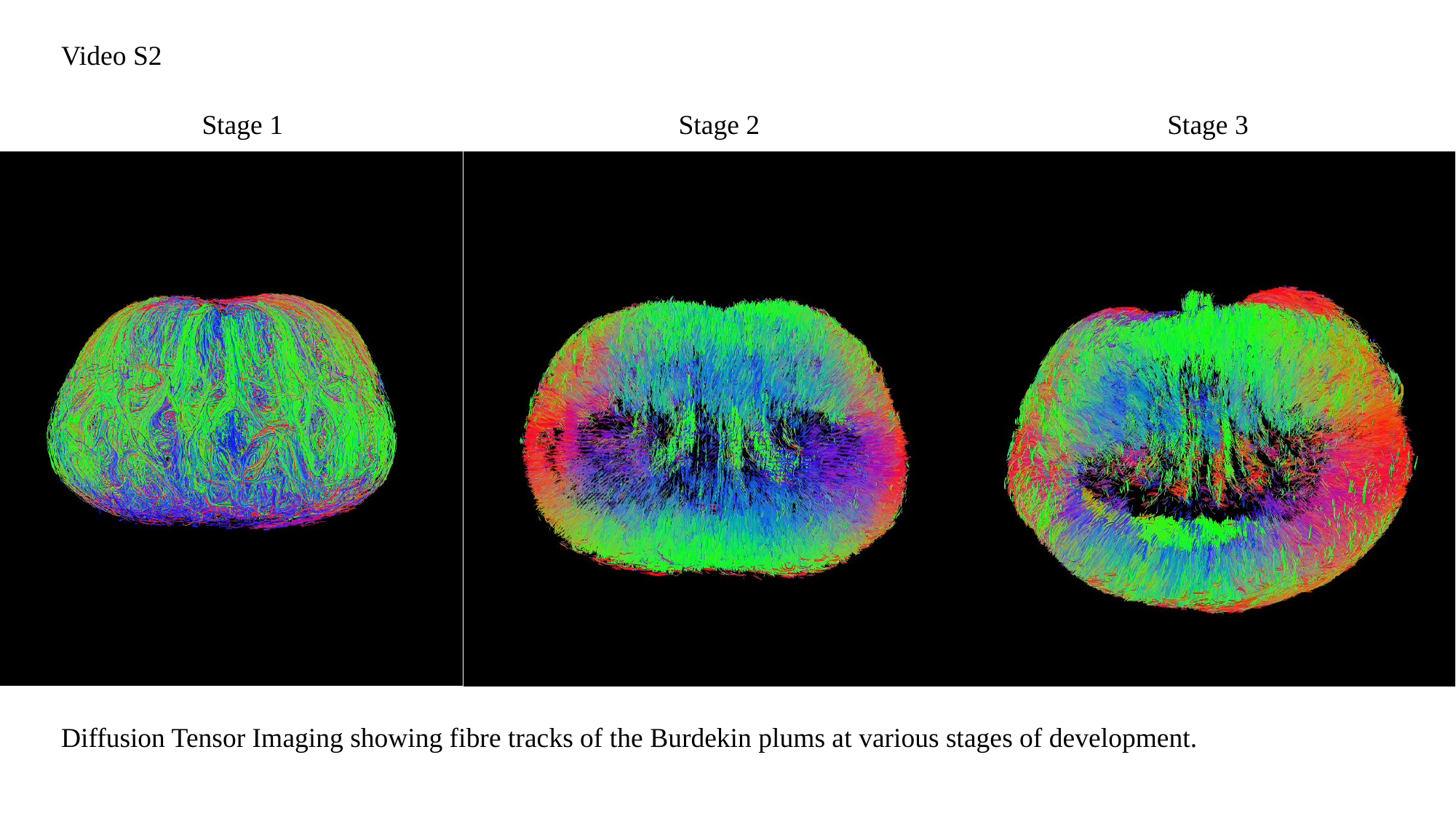

Video S2
Stage 1
Stage 2
Stage 3
Diffusion Tensor Imaging showing fibre tracks of the Burdekin plums at various stages of development.
